# A massive tsunami promoted gene flow and increased genetic diversity in a near threatened plant species

**DOI:** 10.1038/s41598-017-11270-5

**Published:** 2017-09-07

**Authors:** Kako Ohbayashi, Yoshikuni Hodoki, Natsuko I. Kondo, Hidenobu Kunii, Masakazu Shimada

**Affiliations:** 1 Graduate School of Arts and Sciences, The University of Tokyo, Komaba, Meguro, Tokyo, 153-8902 Japan; 2 Department of Biology, Keio University, Hiyoshi, Yokohama, Kanagawa, 223-8521 Japan; 3 Center for Environmental Biology and Ecosystem Studies, National Institute for Environmental Studies, Tsukuba, Ibaraki, 305-8506 Japan; 4 Estuary Research Center, Shimane University, Matsue, Shimane 690-8504 Japan; 5 Present Address: Center for Ecological Research, Kyoto University, Otsu, Shiga, 520-2113 Japan

## Abstract

The magnitude and frequency of disturbances affect species diversity and spatial distributions, but the direct effects of large-scale disturbances on genetic diversity are poorly understood. On March 11, 2011, the Great Tohoku Earthquake in Japan caused a massive tsunami that resulted in substantial alteration of community compositions. Populations of a near-threatened tidal marsh *Carex rugulosa* inhabiting brackish sandbars was also affected. We found four out of six remnant *C. rugulosa* populations along the Pacific Ocean had become completely extinct. Newly emergent post-tsunami populations, however, had higher allelic numbers than pre-tsunami populations, indicating higher genetic diversity after the tsunami. In addition, genetic differentiation (F*st*) between post-tsunami populations was significantly lower than that of pre-tsunami populations. We therefore conclude that the tsunami enhanced gene flow. Seeds of many *Carex* species persist for long periods in soil, which suggests that seed banks are important genetic resources for post-disturbance recovery of genetic diversity. When its brackish sandbar habitat is no longer subject to disturbance and changes to the land, *C. rugulosa* is outcompeted by terrestrial plant competitors and eliminated. Disturbance is a driving force for the recovery and maintenance of populations of species such as *C. rugulosa*—even after near-complete eradication.

## Introduction

The magnitude and frequency of disturbances affect species diversity and spatial distributions^1^. Because a natural disturbance alters the genetic structure of species^2–11^, information of the genetic structures of pre- and post-disturbance populations is required to clarify how the genetic diversity of organisms is affected by disturbance. However, predicting when and where disturbances will occur is generally impossible; thus, samples collected before an extreme disturbance event are rarely available. To understand how disturbances affect genetic diversity, the comparison of pre- and post-disturbance samples is thus valuable and important.

Plant life history strategies are partly determined by their tolerance to disturbance and environmental stresses. Resistance properties are classified as competitive, stress-tolerant, and ruderal, based on the intensity of stress and disturbance^12, 13^. There have been controversies surrounding the issue of why clonal plants have maintained both vegetative and seed reproduction, despite their fitness being increased by vegetative reproduction^14^. Silvertown argued that seed reproduction is more favourable than vegetative reproduction under fluctuating environments^14^; however, this hypothesis has been rarely tested. Seed reproduction enables plants to establish new populations in new habitats via seedling recruitment. Disturbance and seedling recruitment are therefore closely related to spatial and temporal genetic structure. If an undisturbed condition continues following a disturbance, the rate of seedling recruitment within a local population will decline and individuals will expand their habitat via vegetative propagation. Genetic diversity will then decrease with time as a result of clonal propagation or competition between clones in a local population^14^.

**Figure 1:**
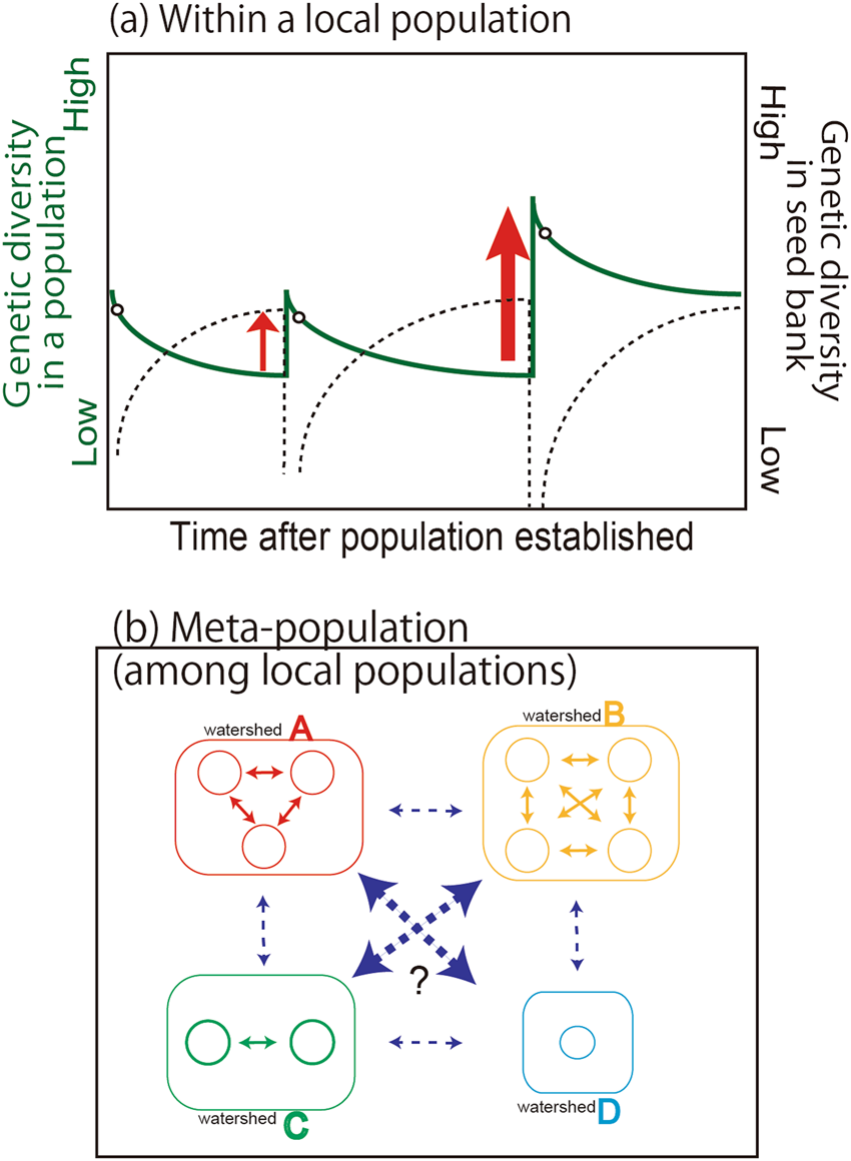
Hypothesised relationships between tsunami and genetic diversity within and among local populations of *C. rugulosa*. (**a**) Within a local population. The x-axis shows elapsed time from the establishment of a population. The left y-axis represents genetic diversity within an above-ground population. The green line indicates increases and decreases in genetic diversity within a local population through time. A white circle on the green line corresponds to the onset of seed production (individual maturation). The right y-axis indicates seed bank genetic diversity and the black dashed line indicates the genetic diversity of seeds maintained in the soil. Red arrows indicate disturbance events that destroy sandbars and create new ones. Disturbance frequency (assuming that a tsunami occurs once every several decades) is indicated by the appearance of red arrows through time. An arrow represents the magnitude of a disturbance. (**b**) The relationship between disturbance and gene flow in the meta-population (among local populations). Squares A to D represent geographically separated watersheds, while circles within squares indicate a presumed population within the watershed. Solid arrows indicate gene flow among populations and dashed arrows represent gene flow by disturbance. A question mark indicates whether the possibility of genetic mixture among distant populations will occur or not.

Here, we hypothesise that how disturbance affect the genetic diversity within and among local populations of *C. rugulosa* (Fig. 1). *C rugulosa* is designated as a near-threatened species on Japanese Red Data List^15, 16^ because existing populations of *C. rugulosa* have decreased in Japan as a result of anthropogenic reclamation of river sandbars. *C. rugulosa* inhabits brackish sandbars and has a low competitive ability^17^. Therefore, this species employs both stress-tolerant (S) and ruderal (R) strategies being classified as an SR strategy following Grim^12, 13^. This species maintains its populations by both sexual (seed) and asexual (clonal) reproduction (new shoots budding from rhizomes). The relationship between wave disturbance and genetic diversity is strongly linked to the temporal characteristics of *C. rugulosa* reproduction: specifically, time to maturity and seed bank longevity^17^. New shoots budding from rhizomes of *C. rugulosa* can produce flowering stems within a year^18^. *Carex* sp. seeds can persist in soil for long periods of time (from 15 to 130 years)^19, 20^ and genets are known to be long-lived (over 100 years)^21^. Therefore, we predict that the above-ground genetic diversity of established populations becomes lower over time in the absence of disturbance because of vegetative propagation by themselves, competition between clones and/or genetic drift^14^ (Fig. 1a). We also have an operational hypothesis that the genetic diversity of soil seed banks before a disturbance, of a near-threatened *C. rugulosa* in Japan, is higher than that of the present above-ground population because the seed banks of previously above-ground extinct populations would have had high genetic diversity. Intensive disturbance, such as massive tsunamis, may play an important role in increasing genetic diversity available from the soil seed bank. We presume that soil seed banks can be released^22^ by a single tsunami, with the amount of released seed depending on the magnitude of the disturbance. The quantities of seeds in soil, which are once reduced rapidly following a tsunami because of germinating or eroding/transporting soil to other places by tsunami waves, are gradually replenished from a local population over time. As shown in Fig. 1a, the increasing disparity in genetic diversity between above-ground populations (green line) and seed banks (black dashed line) just following a tsunami indicate that new genotypes in newly emergent populations accumulate from mutations on the genome. And new genotypes are recruited from neighbouring populations through gene flow caused by tsunami waves which transport floating rhizomes and/or seeds from one watershed to the other (Fig. 1b). The magnitude of wave disturbance may affect the gene pool mixture, recruit new genotypes, and promote gene flow at the metapopulation-level (Fig. 1b). As a result, newly emergent local populations established following a disturbance such as a massive tsunami would, to a great extent, have high genetic diversity and low genetic differentiation because new populations would be established by floating rhizomes and/or seeds through tsunami waves.

**Figure 2:**
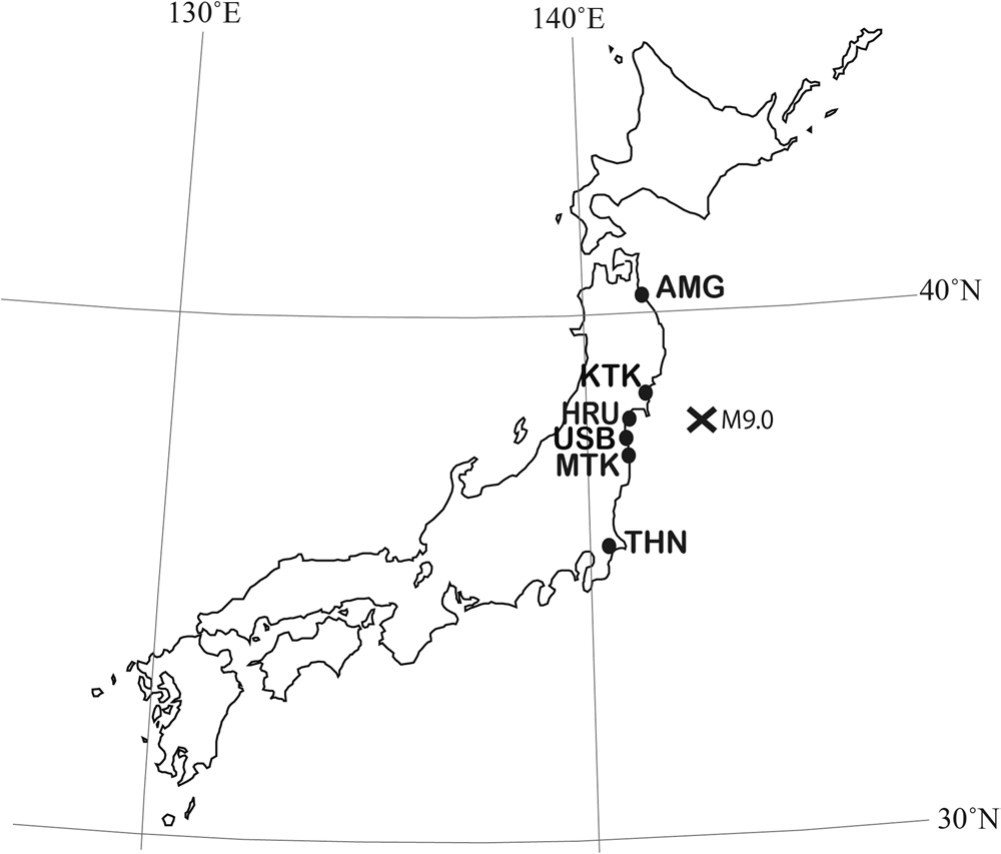
Sampling locations. The cross on the map indicates the epicenter of the March 11, 2011, Tohoku earthquake according to the United States Geological Survey of the United States Department of the Interior (http://earthquake.usgs.gov/earthquakes/eventpage/official20110311054624120_30#executive). Population abbreviations are defined in Supplementary Table S1. The map was drawn by the author using Adobe Illustrator CS6 with reference to Google Maps (©Google, https://www.google.co.jp/maps/).

The Great Tohoku Earthquake (magnitude 9.0 [M_w_]) in Japan, the fourth largest earthquake in the world since 1900, also caused a giant tsunami^23^ that resulted in substantial alteration of biological community compositions^24–26^. In the northeast Honshu coastal region (N36° to N40°; Fig. 2), massive tsunamis caused by large earthquakes have been recorded five times during the past 123 years^27^ (1894, 1896, 1933, 1960, and 2011). The 2011 tsunami destroyed approximately 500 km of coastline, from north to south, in the northeast Honshu. In 2008 (hereafter, pre-tsunami), populations of *C. rugulosa* inhabited only six sites along the Pacific Ocean coast of Japan (northeast Honsyu)^28^. We therefore investigated all these six populations in 2013 or 2014 (hereafter, post-tsunami).

In this study, we tested the hypothesised relationship between a single, extreme massive wave disturbance and genetic diversity within and among local populations (Fig. 1) using the clonal tidal sedge *C. rugulosa*. The genetic composition of pre- and post-tsunami populations was assessed using microsatellite markers and the result showed increasing genetic diversity, and gene flow increased while fixation indexes (F*st*) within and among populations decreased after the tsunami.

## Results

### Pre- and post-tsunami sampling sites

Samples were collected in 2008^28^, before the tsunami, and after the tsunami in 2013 and 2014 (Fig. 2). The most northern population (AMG) was affected by the earthquake and the tsunami, having experienced a tsunami run-up height of 2.3 m^29^. This population survived and was still existing at the same location in 2014; thus, several genet samples from AMG were shared pre- and post-tsunami. From KTK to MTK populations were severely affected by the earthquake and tsunami and all local populations of above-ground plants and sandbars completely eliminated. However, newly created sandbars were formed by the tsunami, with new populations found in each watershed. We classified these new populations as post-tsunami populations. The most southern population (THN) was affected by the earthquake and then extinguished by anthropogenic river improvement. Therefore, neighbouring populations were collected in 2014. A rough estimate of distances between sampling sites of pre-and post-tsunami populations within the same watershed and the tsunami run-up height at each site are given in Supplementary Table S1.

### Population genetic analysis

**Figure 3:**
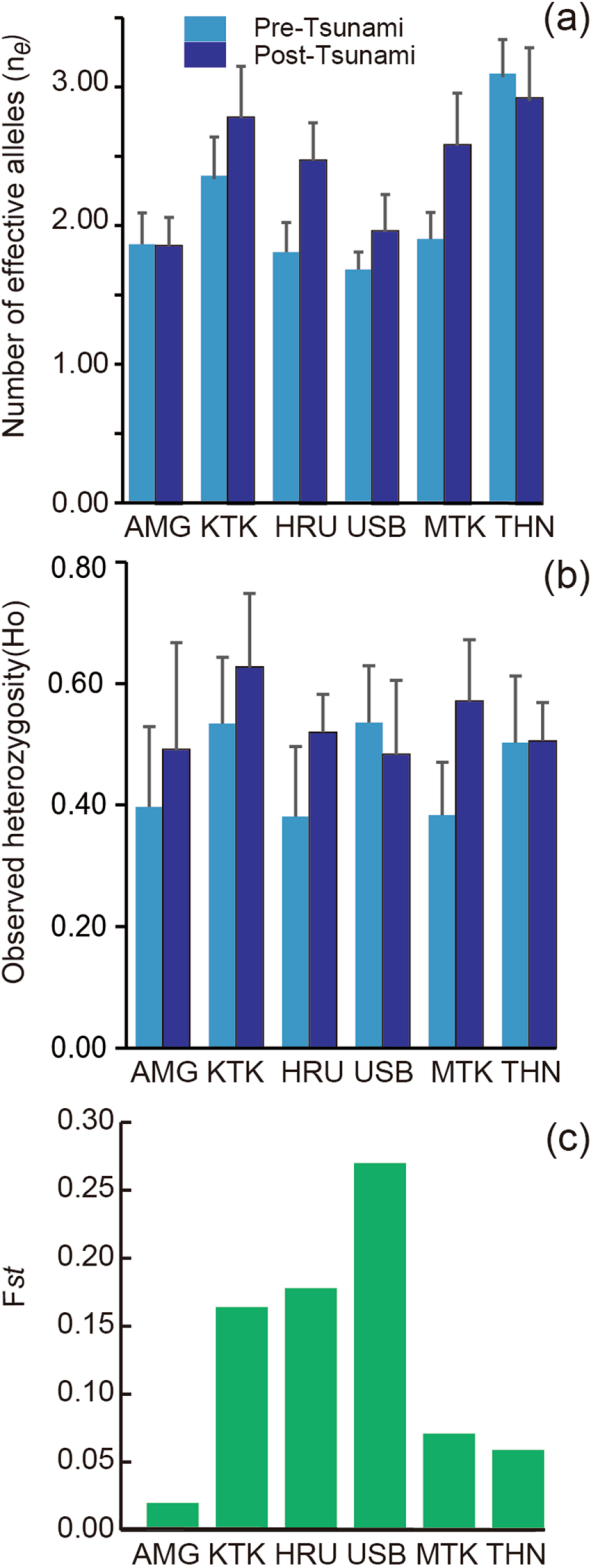
Genetic diversity parameters of pre- and post-tsunami samples. The population AMG was not included in the statistical analysis (a and b, see details in Methods). (**a**) The number of effective alleles (Mean ± Standard Error, *p* < 0.01, bootstrap test). (**b**) Observed heterozygosity (Mean ± Standard Error, *p* < 0.001, bootstrap test). (**c**) F*st* values within the same watershed pre- vs. post-tsunami. Population locations and abbreviations are the same as in Fig. 2 and Supplementary Table S1.

Post-tsunami populations had significantly more effective alleles than pre-tsunami populations (*p* < 0.01, bootstrap test; Fig. 3a and Supplementary Table S1). Levels of observed heterozygosity were significantly different pre- vs. post-tsunami populations (*p* < 0.001, bootstrap test; Fig. 3b and Supplementary Table S1). Coefficients of genetic differentiation (F*st*) within the same watershed were calculated pre- vs. post-tsunami local populations. If genetic composition was unchanged pre- vs. post-tsunami, the F*st* of 0 would be obtained. Except for surviving AMG, all populations had F*st* values greater than 0.05. Analysis of molecular variance (AMOVA) revealed that the following percentages of total microsatellite variation occurred pre- vs. post-tsunami at each site; AMG 1.8%, KTK 16.3%, HRU 18.2%, USB 25.9%, MTK 7.2%, and THN 6.3% (Fig. 3c and Supplementary Table S2a). Except for AMG, relatively large genetic variation (%) were observed. Thus, the genetic makeup of newly created post-tsunami populations was shaped by the tsunami.

In two randomly selected post-tsunami local populations, genotypic compositions within a local population, whether due to seed recruitment or clonal propagation after the tsunami, were evaluated. One of the HRU local populations had only a single genotype (Supplementary Fig. S1a and b) while the MTK population had eight genotypes (Supplementary Fig. S1c).

**Figure 4:**
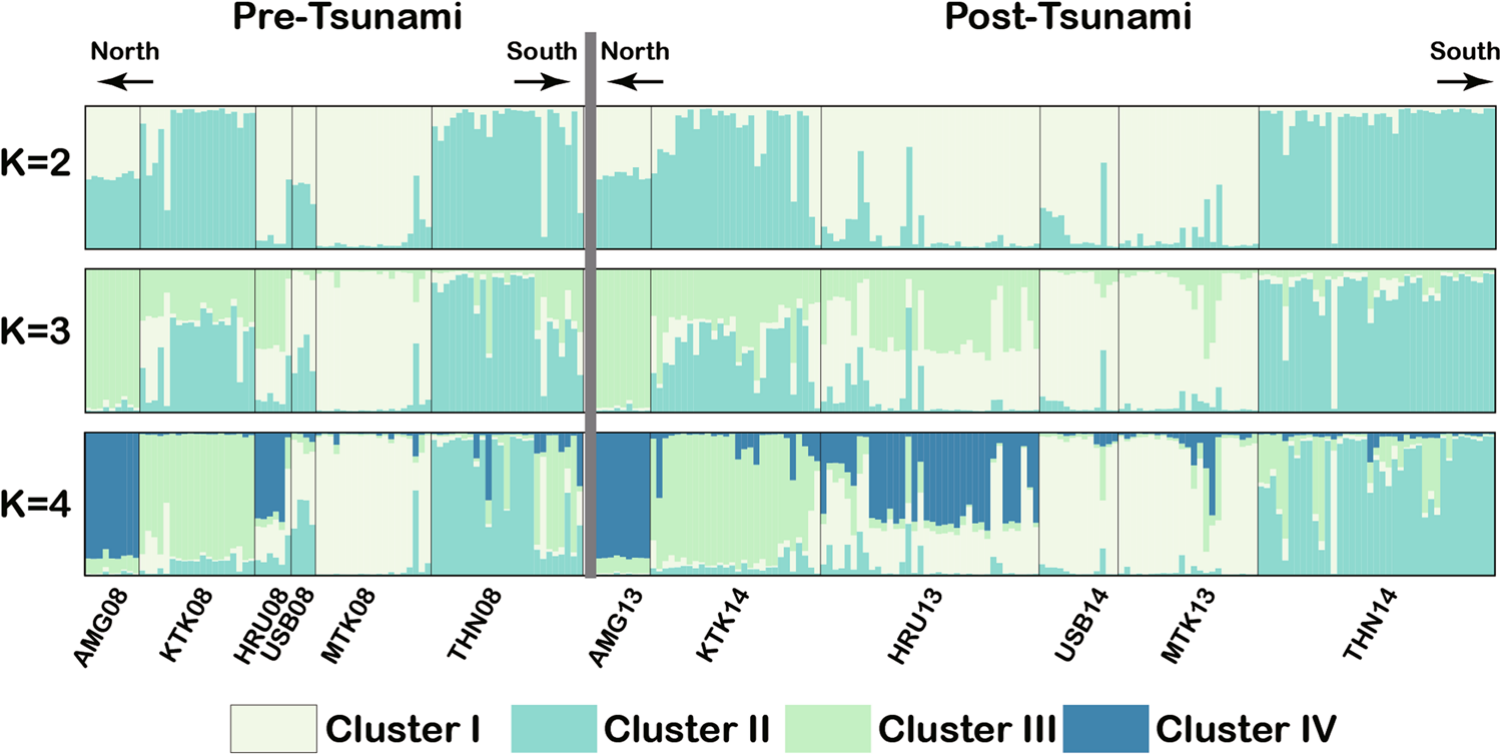
Genetic structure in pre- and post-tsunami populations inferred using STRUCTURE. Different coloured bars represent different clusters. The optimal value of *K* was 2, with results obtained using *K* = 3 and 4 also shown. Population locations and abbreviations are given in Fig. 2 and Supplementary Table S1. For details of calculations, see Methods.

A STRUCTURE analysis revealed two clusters (*K* = 2) whose genetic composition was largely similar pre- and post-tsunami samples (Fig. 4). For data comparison across plausible *K* values, results for *K* = 3 and 4 are also shown in Fig. 4 ^30^. Interestingly, clustering tendencies were not so different pre- vs. post-tsunami populations. At *K* = 3, relatively distinct cluster differences were observed among pre-tsunami sites, whereas the post-tsunami results revealed gradual admixture between neighbouring populations such as KTK and HRU, and USB and MTK.

**Figure 5:**
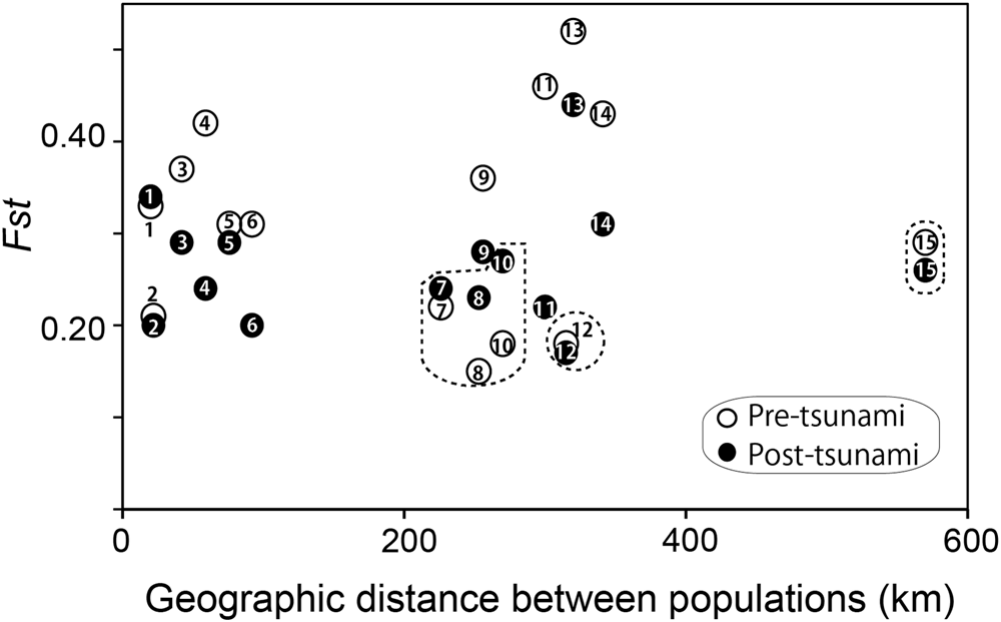
Changes in genetic differentiation (fixation index, F*st*) pre- vs. post-tsunami populations. The x-axis shows geographic distances between populations, while the y-axis indicates F*st*. Numbers in white and black circles represent pre- and post-tsunami F*st* values, respectively, based on the following comparisons: 1, HRU vs. USB; 2, USB vs. MTK; 3, HRU vs. MTK; 4, KTK vs. HRU; 5, KTK vs. USB; 6, KTK vs. MTK; 7, MTK vs. THN; 8, USB vs. THN; 9, AMG vs. KTK; 10, HRU vs. THN; 11, AMG vs. HRU; 12, KTK vs. THN; 13, AMG vs. USB; 14, AMG vs. MTK; and 15, AMG vs. THN. Dashed lines represent F*st* combination with THN. Population locations and abbreviations are shown in Fig. 2 and Supplementary Table S1. Geographic distances between populations and AMOVA results are reported in Supplementary Table S2.

Notably, F*st* values were significantly lower in post-tsunami (F*st* = 0.18) than pre-tsunami (F*st* = 0.29) populations (Fig. 5 and Supplementary Table S2c and e). AMOVA confirmed that the genetic variance among post-tsunami populations (18.1%) was lower than that among pre-tsunami ones (28.6%). F*st* decreased significantly in the pair-wise F*st* values in post-tsunami (the paired-sample t-test, t = 2.19, *df* = 14, p = 0.046). As tendencies of THN showed differences from other watersheds, F*st* combinations with THN were circled by dashed lines (Fig. 5). The result of a binomial test pre- vs. post-tsunami using all F*st* values including THN showed no significant difference (p = 0.549). However, after eliminating THN, post-tsunami F*st* decreased significantly (p = 0.039). For HRU vs USB (Fig. 5, no.1), the difference may be caused by the low sample size of the pre-tsunami USB population.

## Discussion

The tsunami created new tidal marsh habitats, which allowed newly established *C. rugulosa* populations to increase their genetic diversity and lowered genetic differentiation by promoting gene flow. These results support our operational hypothesis illustrated in Fig. 1a. A single disturbance enhances genetic diversity in clonal sedge. The phenomenon of increased genetic diversity after the disturbance has been reported previously^7, 8^. Our assessments of gene flow revealed gradual mixing between geographically neighbouring populations, but no drastic interchange among relatively distant populations. Therefore, our hypothesis presented in Fig. 1b is partly supported but does not apply to mixed distant populations.

A binomial test was conducted both, including THN or without THN in F*st* results in Fig. 5. This was needed because even though the Tone River (THN) was affected by the Great Tohoku Earthquake and the tsunami, the tsunami did not transport sands or create new sandbars in this watershed because of its distance from the epicentre (Fig. 2). Furthermore, THN local populations grew on the riverbanks utilised as parks and small harbours. We could not collect enough samples of THNa and THNb in 2013, because the riverbank protection works reduced population size (THNa) or almost eliminated the population (THNb). Therefore, as THN local populations were affected by anthropogenic works, tendencies would be different from other watersheds where the tsunami completely destroyed populations and created new sandbars.

The slight decline in genotypic diversity (*R*) and the similar effective number of alleles pre- vs. post-tsunami AMG population appears to be due to vegetative propagation over five years, as several genets were shared between 2008 and 2014 samples. Nevertheless, the higher genetic diversity of other populations following the tsunami suggests that new populations were established by floating rhizomes and/or seeds. As the tsunami struck coastlines at the end of the winter, most above-ground plants had already died and some of the rhizomes may have died. We therefore postulate that many populations after tsunami were established by seeds. We also hypothesise that the observed increase in genetic diversity within watersheds was due to soil seed banks^19, 20, 22, 31, 32^. The increase in the effective number of alleles per locus following the tsunami suggests the introduction of new alleles into populations. Seed banks thus likely played an important role in the restoration of genetic diversity within local populations following the massive tsunami. This suggestion is in agreement with the idea that seed reproduction rather than clonal propagation is enhanced under fluctuating conditions^14^. The spatial genetic composition of two local populations showed two interesting tendencies; one consisting of only one genotype (a clone), the other consisting of eight genotypes (Supplementary Figure S1). Even though we couldn’t clarify which factor, rhizomes or seeds, established those populations, there were two types of genetic composition extant in the same period after the tsunami. Further study is needed to clarify which factor provoked the onset of population establishment.

Even if above-ground populations of *C. rugulosa* a near-threatened species in Japan^15, 16^ become extinct, a seed bank can remain in a sandbar and lead to the establishment of new colonies. In previous studies, the germination of *C. rugulosa* seeds was found to rely strongly on light induction^19, 20^. When an above-ground population (mainly leaves) shows high density, the creation of generational alterations via seed germination is difficult because of the limited light access. Seeds of many *Carex* species are not only buoyant and suitable for water dispersal, but can also persist in the soil for long periods of time^19, 20^ and these specific traits of *Carex* seeds may thus have also promoted extensive gene flow following the tsunami. Although some tidal marsh species such as *C. rugulosa* are overwhelmed by terrestrial plant competitors under undisturbed conditions^18^, they can nonetheless maintain themselves and increase genetic diversity via a single disturbance—even if such disturbance almost completely destroys their populations. We acknowledge that different locations pre- and post-tsunami were a necessary limitation because of the destruction of land by the tsunami and that our field surveys were limited to only one pre- and post-tsunami sampling. However, the results of the data showed consistencies in the same general direction; increasing genetic diversity and decreasing fixation indexes. Therefore our findings strongly suggest that the tsunami would have had an important role in the maintenance of the near-threatened salt marsh populations.

## Methods

### Target organism and sample collection

*C. rugulosa* is distributed throughout East Asia^33^. To clarify this species’ conservation management unit in Japan, we studied 22 populations in 2008 and analysed the genetic structure within and among populations using microsatellite markers^28, 34^. Of the 22 populations, six were along the Pacific Ocean coast; therefore, the pre- and post-tsunami genetic compositions of those six populations were compared in this study. We visited the Tohoku region in 2013 and 2014. When we found a *C. rugulosa* population, we roughly estimated its area using a measuring tape to determine its dimensions. For DNA collection, 5-cm-long leaves were collected at about 0.5 m to 1 m intervals along with the longest axis of each population. The number of ramets and flowering stems (May 2013) was counted within 10 plastic quadrats (20 cm × 20 cm). The quadrats were randomly positioned within each population. The total number of ramets per population was roughly estimated using the mean ramet density. Samples were kept at −20 °C until DNA extraction.

### DNA extraction

Plant DNA was extracted from homogenised frozen leaves using a DNeasy Plant Mini kit (Qiagen, Hilden, Germany) following the manufacturer’s instructions.

### DNA amplification and fragment analysis

Seven microsatellite loci were assessed, namely CR5, 7, 10, 21, 27, 28, and 39, under PCR conditions slightly modified from previous reports^28, 34^ with annealing temperatures (Tm) of 57, 57, 62, 57, 62, 57, and 62 °C, respectively. We amplified DNA samples in 10 µL reaction mixtures containing 1 µL template DNA, 1 × PCR buffer for KOD FX NEO, 0.4 mM of each dNTP, 1 U of KOD FX NEO polymerase (Toyobo, Osaka, Japan), 0.5 µM fluorescently tagged forward primer (6-FAM, NED, Applied Biosystems), and 0.5 µM reverse primer. PCR parameters were as follows: a 2-min hot start at 94 °C, followed by 35 cycles of 10 s at 98 °C, 30 s at the appropriate Tm, and 1 min at 68 °C. The amplified DNA was analysed on an ABI 3130xl or ABI 3730xl DNA analyser (Applied Biosystems). Allele sizes were assessed by fragment analysis. To correct for the influence of DNA analyser differences between samples from 2008 and samples from this study, samples from the Ohashi River in Shimane Prefecture previously analysed by Ohbayashi *et al*.^28, 34^ were reanalysed and corrected for allele size to enable direct comparison of allele sizes between the previous (pre-tsunami) samples and those in this study (post-tsunami individuals).

### Data analysis

Data from six pre-tsunami^28^ and post-tsunami populations along the Pacific Ocean in northeast Honshu were used. Pre-tsunami data from INS, THNa, and THNb populations, which were distributed within the Tone River watershed, were pooled and collectively designated as the pre-tsunami THN08 population for comparison with post-tsunami THN14 populations in all analyses. Approximate distances among these three pre-tsunami population were as follows: INS (furthest upstream) to THNa, 30 km; THNa to THNb (furthest downstream), 3 km; INS to THNb, 33 km. We were unable to access the INS population following the tsunami because it is located in a restricted area established by the Ministry of Environment for plant conservation. Because THNa and THNb population size had reduced due to anthropogenic works, we collected two additional neighbouring populations within 5 km distance in the Tone River watershed. All analyses were performed using only a distinct multilocus genotype (a genet) to eliminate the effects of clonal individuals on allele frequency at each locus (a ramet)^35^. GENECLONE software^36^ was used to estimate clonal genotypic diversity parameters, namely, number of distinct and identical multilocus genotypes (genets, N_G_), as well as genotypic diversity (clonal diversity) calculated as *R* = (*G* − 1)/(*N* − 1), where *G* is the number of distinct genotypes and *N* is the number of genotyped samples. We also calculated observed (H*o*) and expected (H*e*) heterozygosities using CERVUS software^37^ (version 3.0.6). The effective number of alleles per locus (n_*e*_) was also calculated. Heterozygote excess or deficiency within a population was tested using a Markov-chain algorithm developed by Guo and Thompson^38^ and implemented in the software program GENEPOP^39^ version 4.2. The inbreeding coefficient (F*is*) and pairwise population differentiation among populations (F*st*) were also calculated and an unbiased estimate of the *P*-value of a log likelihood ratio (G)-based exact test was performed on F*st* using GENEPOP. Although the pre-tsunami USB population consisted of only four genets which were an insufficient sample size for F*st* calculation, we included pre-tsunami USB in our F*st* calculations to know rough tendencies in this study. Bonferroni corrections were applied to all significant pairwise-test results to adjust for multiple comparisons. The probability of identity (PI)—which is the probability that two individuals obtained at random from a population would have the same multilocus genotype^40^ —was also calculated.

Individual-based Bayesian clustering methods implemented in STRUCTURE^41^ were used to estimate pre- vs. post-tsunami population genetic structures (226 genets, 11 populations). We selected the admixture model and the F model (correlated allele frequencies) without a priori information. STRUCTURE uses a Bayesian clustering method to assign each multilocus genotype to one of *K* probable cluster populations. For each *K* value from 1 to 10, we performed 20 independent runs of 1 × 10^5^ iterations after a burn-in period of 1 × 10^5^ iterations. To detect the true number of clusters, we then calculated Δ*K*, which is based on the rate of change in posterior probability values for *K* (log probability of data; L [*K*]) between successive *K* values^42^. The 20 runs performed with the selected *K*-value were retained. Replications of STRUCTURE runs were combined using the software programs STRUCTURE HARVESTER^43^ and CLUMPP^44^. For *K* = 2, the FullSearch algorithm in CLUMPP was used to align runs. For *K* = 3 and 4, we used the Large K-Greedy algorithm, with 1 × 10^6^ random input orders. Results were visualised using the software program DISTRUCT^45^.

### Statistical analysis

The F*st* differences among populations pre- vs. post-tsunami samples were analysed using the two-tailed paired-sample t-test^46^ and the binomial test^46^ in R version 3.3.1. For other genetic parameters (clonal diversity [*R*], observed heterozygosity (H*o*), the number of effective alleles (n_*e*_)), bootstrap analyses^47^ were performed to test the differences (residual) for each parameter pre- vs. post-tsunami samples. For this calculation, the AMG population was excluded since the population survived after the tsunami and shared genets pre- and post-tsunami. Mean pairwise differences between the same population in *T* pre- and post-tsunami sites (where *T* is the number of observed sampling sites) formed by random sampling from pooled observed data were used as the test statistic. This generated a probability distribution of pre- and post-tsunami population differences under the null hypothesis that there was no mean difference pre- vs. post-tsunami values. The bootstrap test was performed on each parameter until T random combinations of 9999 sets were achieved, following which the observed mean pairwise difference was placed against the probability distribution. If the observed value fell within the upper or lower 250 ranks of the 10000 sets in the parameter (0.025%), we then confirmed that there was a significant pre- vs. post-tsunami difference using a two-tailed test. To estimate genetic variation and genetic differentiation (fixation index) pre- vs. post-tsunami populations, AMOVA was performed using Arlequin^48^ version 3.5. The null hypothesis was no genetic differentiation among populations exists (F*st* = 0), while the working hypothesis was that F*st* values are significantly larger than 0 (F*st* ≠ 0).

## Electronic supplementary material

Supplementary figures and tables
