# Supplementary material for: A massive tsunami promoted gene flow and increased genetic diversity in a near threatened plant species
Source: Sci Rep. 2017 Sep 7;7:10933. doi: 10.1038/s41598-017-11270-5 (PMC5589756; doi:10.1038/s41598-017-11270-5)
Supplement: Supplementary file 1 — Supplementary figures and tables [file 41598_2017_11270_MOESM1_ESM.doc]

**Supplementary information**

**Title: A massive tsunami promoted gene flow and increased genetic diversity in a near threatened plant species**

**Authors**: Kako Ohbayashi^1,5^, Yoshikuni Hodoki ^2,5^, Natsuko Kondo^3^, Hidenobu Kunii ^4^, Masakazu Shimada ^1^

**Affiliations :** ^1^ Graduate School of Arts and Science, The University of Tokyo, 3-8-1 Komaba, Meguro, Tokyo 153-8902, Japan; ^2^ Department of Biology, Keio University, 4-1-1 Hiyoshi, Kohoku-Ku, Yokohama-shi, Kanagawa 223-8521, Japan; ^3^ Center for Environmental Biology and Ecosystem Studies, National Institute for Environmental Studies, Tsukuba 305-8506, Japan;^4^ Research Center for Coastal Lagoon Environments, Shimane University, Matsue, Shimane 690-8504, Japan

**Present address**: ^5^ Center for Ecological Research, Kyoto University, Hirano, Otsu, Shiga 520-2113. kakoh@ecology.kyoto-u.ac.jp

**
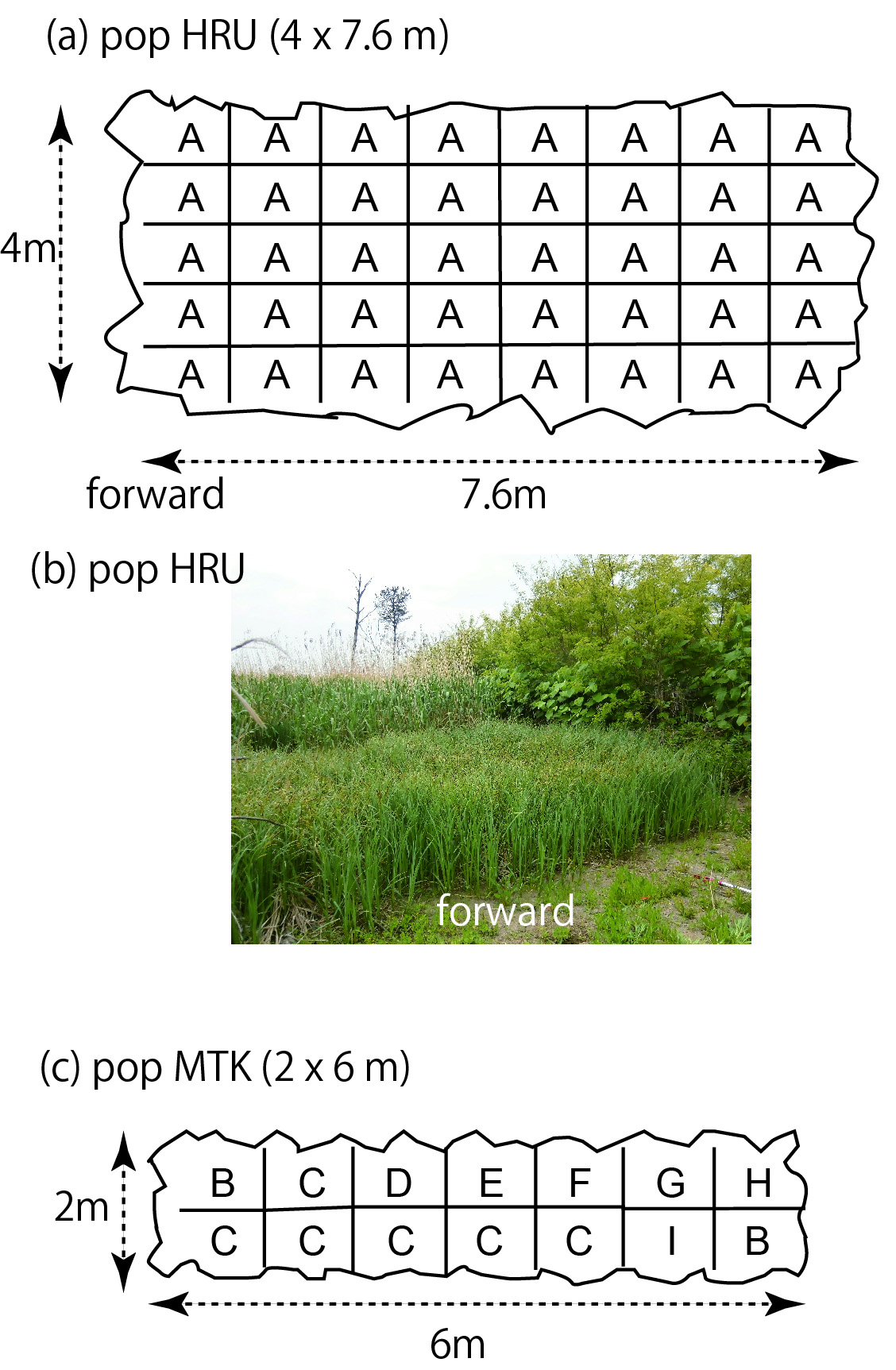
**

**Supplementary Figure S1.**Spatial genotypic composition within a population. Capital letters represent the same genotype. (a) One of HRU population; N = 40; the number of genotypes =1, (b); a photograph of HRU population (May 2013). (c) MTK population; N =14; the number of genotypes = 8.

**Supplementary Table S1.** Results of genetic analyses of pre- and post-tsunami populations of *Carex rugulosa* along the northeast Honshu coast. Sampling date, pre-tsunami (2008, cited from Ohbayashi et al., 2012 ) or post-tsunami (2013 or 2014, this study); Status, status after the tsunami; surviving, population survived at the same place; PD, new, Population was destroyed, but new populations were established; Tsunami, tsunami run-up height at the location^29^, with an asterisk indicating tsunami inundation height^29^ ; Distance, rough estimation of sampling distances pre- vs post-tsunami population within the same watershed; # of local pop, number of local populations within a watershed; Area, cumulative habitats within a watershed; Flowering, percentages of flowering stems within a habitat area during the flowering season (late May to early June); *N*, number of samples examined (ramets); N_G_, number of identical genotypes (genets); *R*, genotypic richness (clonal diversity, [*R*]= [G-1] / [N-1]); H*_O_*, observed heterozygosity; H*_E_*, expected heterozygosity; n*_e_*, effective number of alleles per locus; F*_IS_*, mean inbreeding coefficient; PI, probability of identity across seven loci. *, significant excess or deficiency in heterozygotes within the population (after Bonferroni correction).

| Population | Location | Sampling date | Tsunami | Status | Distance | # of local pop | Area (m^2^) | Density m^-2^ | Flowering (%) | N | *N_G_* | *R* | *Ho* | *He* | *n_e_* | *F*_IS_ | PI |
| --- | --- | --- | --- | --- | --- | --- | --- | --- | --- | --- | --- | --- | --- | --- | --- | --- | --- |
| AMG | Amagamori, Takase River, Aomori | May 2008 |  |  |  | - | 600 | 115 | 21.7 | 21 | 9 | 0.40 | 0.40 | 0.40 | 1.85 | -0.10 | 0.0017 |
|  |  | May 2013 | 3.6m | Surviving | 0 m | - | 600 | 108 | 7.7 | 33 | 9 | 0.25 | 0.49 | 0.41 | 1.87 | -0.15 | 0.0020 |
| KTK | Kitakami River, Miyagi | May 2008 |  |  |  | - | 20000 | 83 | 15.2 | 21 | 19 | 0.90 | 0.53 | 0.52 | 2.35 | -0.06 | 0.0001 |
|  |  | Oct 2014 | 13.7m | PD, new | 2.2 km | - | 36 | 55 | - | 30 | 28 | 0.93 | 0.63* | 0.56 | 2.79 | -0.10 | <0.0001 |
| HRU | Natori River, Miyagi | June 2008 |  |  |  | - | 18 | 238 | 10.5 | 17 | 6 | 0.31 | 0.38 | 0.43 | 1.80 | -0.14 | 0.0021 |
|  |  | May 2013 | 7.4m* | PD, new | 300m | 11 | 543 | 233 | 35.8 | 108 | 36 | 0.33 | 0.52 | 0.56 | 2.48 | 0.09 | <0.0001 |
| USB | Ushibashi River, Miyagi | June 2008 |  |  |  | - | 14 | 425 | 7.6 | 15 | 4 | 0.21 | 0.54 | 0.38 | 1.67 | -0.27 | 0.0068 |
|  |  | Oct 2014 | 10.3m* | PD, new | 400m | 2 | 20 | 29 | - | 23 | 13 | 0.52 | 0.48* | 0.42 | 1.97 | -0.12 | 0.0008 |
| MTK | Matukawaura, Fukushima | June 2008 |  |  |  | - | 150 | 220 | 5.7 | 20 | 19 | 0.95 | 0.38* | 0.44 | 1.89 | 0.14 | 0.0007 |
|  |  | May 2013 | 21.0m | PD, new | 4.2 km | 3 | 26 | 125 | 40.0 | 32 | 23 | 0.71 | 0.57 | 0.55 | 2.60 | -0.03 | <0.0001 |
| THN | INS, Tone River, Chiba | June 2008 |  |  | see Method | - | 183 | 83 | 15.2 | 16 | 5 | 0.27 | 0.53* | 0.37 | 1.69 | -0.70 | - |
|  | THNa, Tone River, Chiba | July 2008 |  |  |  | - | - | - | - | 14 | 9 | 0.62 | 0.46* | 0.63 | 2.49 | -0.13 | - |
|  | THNb, Tone River, Chiba | July 2008 |  |  |  | - | - | - | - | 20 | 11 | 0.53 | 0.42* | 0.58 | 2.55 | 0.25 | - |
|  | Mean three populations (INS, THNa &THNb) | July 2008 |  | Recalculation for this study | | | |  |  | 50 | 25 | 0.50 | 0.50* | 0.66 | 3.08 | 0.25 | <0.0001 |
|  | Tone River, Chiba | May 2013 | 3.2m | - | within 5km | 4 | - | - | - | 81 | 39 | 0.48 | 0.51* | 0.62 | 2.93 | 0.19 | <0.0001 |

**Supplementary Table S2.** Pairwise F*st* values and AMOVA results among six populations.

**(a)** AMOVA results in pre- vs. post-tsunami population within the same watershed.

| Differences pre- vs. post-tsunami | Source of Variation | d.f. | SSD | Variance components | % Total | Fixation index | P value |
| --- | --- | --- | --- | --- | --- | --- | --- |
| AMG | Among populations | 1 | 1.97 | 0.03 | 1.8 | F*st*=0.02 | p<0.0001 |
|  | Within populations | 34 | 50.30 | 1.49 | 98.2 |  |  |
| KTK | Among populations | 1 | 18.93 | 0.38 | 16.3 | F*st*=0.16 | p<0.0001 |
|  | Within populations | 92 | 177.57 | 1.93 | 83.7 |  |  |
| HRU | Among populations | 1 | 10.71 | 0.43 | 18.2 | F*st*=0.18 | p<0.0001 |
|  | Within populations | 82 | 157.80 | 1.92 | 81.8 |  |  |
| USB | Among populations | 1 | 7.97 | 0.53 | 25.9 | F*st*=0.26 | p<0.0001 |
|  | Within populations | 32 | 48.30 | 1.51 | 74.1 |  |  |
| MTK | Among populations | 1 | 7.43 | 0.14 | 7.2 | F*st*=0.07 | p<0.0001 |
|  | Within populations | 82 | 144.19 | 1.76 | 92.8 |  |  |
| THN | Among populations | 1 | 11.50 | 0.15 | 6.3 | F*st*=0.06 | p<0.0001 |
|  | Within populations | 126 | 238.85 | 2.25 | 93.7 |  |  |

**(b)** Geographic distance among populations (km, upper diagonal), and pre-tsunami pairwise F*st* values among six populations (lower diagonal). Significance levels are as follows: **p* < 0.05; ***p* < 0.01 (G-test after Bonferroni correction).

|  | Pre-tsunami | | | | | |
| --- | --- | --- | --- | --- | --- | --- |
|  | AMG | KTK | HRU | USB | MTK | THN |
| AMG | - | 256 | 300 | 320 | 341 | 570 |
| KTK | 0.36** | - | 59 | 76 | 92 | 315 |
| HRU | 0.46** | 0.42** | - | 20 | 42 | 270 |
| USB | 0.52** | 0.31** | 0.33** | - | 22 | 253 |
| MTK | 0.43** | 0.31** | 0.37** | 0.21** | - | 226 |
| THN | 0.29** | 0.18** | 0.18** | 0.15** | 0.22** | - |

**(c)** AMOVA results among pre-tsunami populations.

| Grouping | Source of Variation | d.f. | SSD | Variance components | % Total | Fixation index | P value |
| --- | --- | --- | --- | --- | --- | --- | --- |
| Pre-Tsunami | Among populations | 5 | 103.1 | 0.75 | 28.6 | F*st*=0.29 | p<0.0001 |
|  | Within populations | 158 | 290.3 | 1.84 | 71.4 |  |  |

**(d)** Post-tsunami pairwise F*st* values among the six populations. Significance levels are as follows: **p* < 0.05; ***p* < 0.01 (G-test after Bonferroni correction).

|  | Post-tsunami | | | | |
| --- | --- | --- | --- | --- | --- |
|  | AMG | KTK | HRU | USB | MTK |
| AMG | - |  |  |  |  |
| KTK | 0.28** | - |  |  |  |
| HRU | 0.22** | 0.24** | - |  |  |
| USB | 0.44** | 0.29** | 0.34** | - |  |
| MTK | 0.31** | 0.20** | 0.29** | 0.20** | - |
| THN | 0.26** | 0.17** | 0.27** | 0.23** | 0.24** |

(e) AMOVA results among post-tsunami populations.

| Grouping | Source of Variation | d.f. | SSD | Variance components | % Total | Fixation index | P value |
| --- | --- | --- | --- | --- | --- | --- | --- |
| Post-Tsunami | Among populations | 5 | 113.2 | 0.44 | 18.1 | F*st*=0.18 | p<0.0001 |
|  | Within populations | 290 | 572.1 | 1.97 | 81.9 |  |  |
